# Supplementary material for: Intraspecific Comparative Analysis Reveals Genomic Variation of Didymella arachidicola and Pathogenicity Factors Potentially Related to Lesion Phenotype
Source: Biology (Basel). 2023 Mar 21;12(3):476. doi: 10.3390/biology12030476 (PMC10045276; doi:10.3390/biology12030476)
Supplement: Supplementary file 1 [file biology-12-00476-s001.zip › Supplementary Figures.pdf]

Supplementary Figures

Intraspecific Comparative Analysis Reveals Genomic Variation of *Didymella arachidicola* and Pathogenicity Factors Potentially Related to Lesion Phenotype

Shaojian Li *,†, Zhenyu Wang *,†, Meng Gao, Tong Li, Xiaowei Cui, Junhuai Zu, Suling Sang, Wanwan Fan and Haiyan Zhang

Institute of Plant Protection, Henan Key Laboratory of Crop Pest Control, International Joint Research Laboratory for Crop Protection of Henan, Key Laboratory of Integrated Pest Management on Crops in Southern Region of North China, Henan Academy of Agricultural Sciences, Zhengzhou 450000, China

* Correspondence: lishaojianli@126.com (S.L.); wangzy21@163.com (Z.W.)

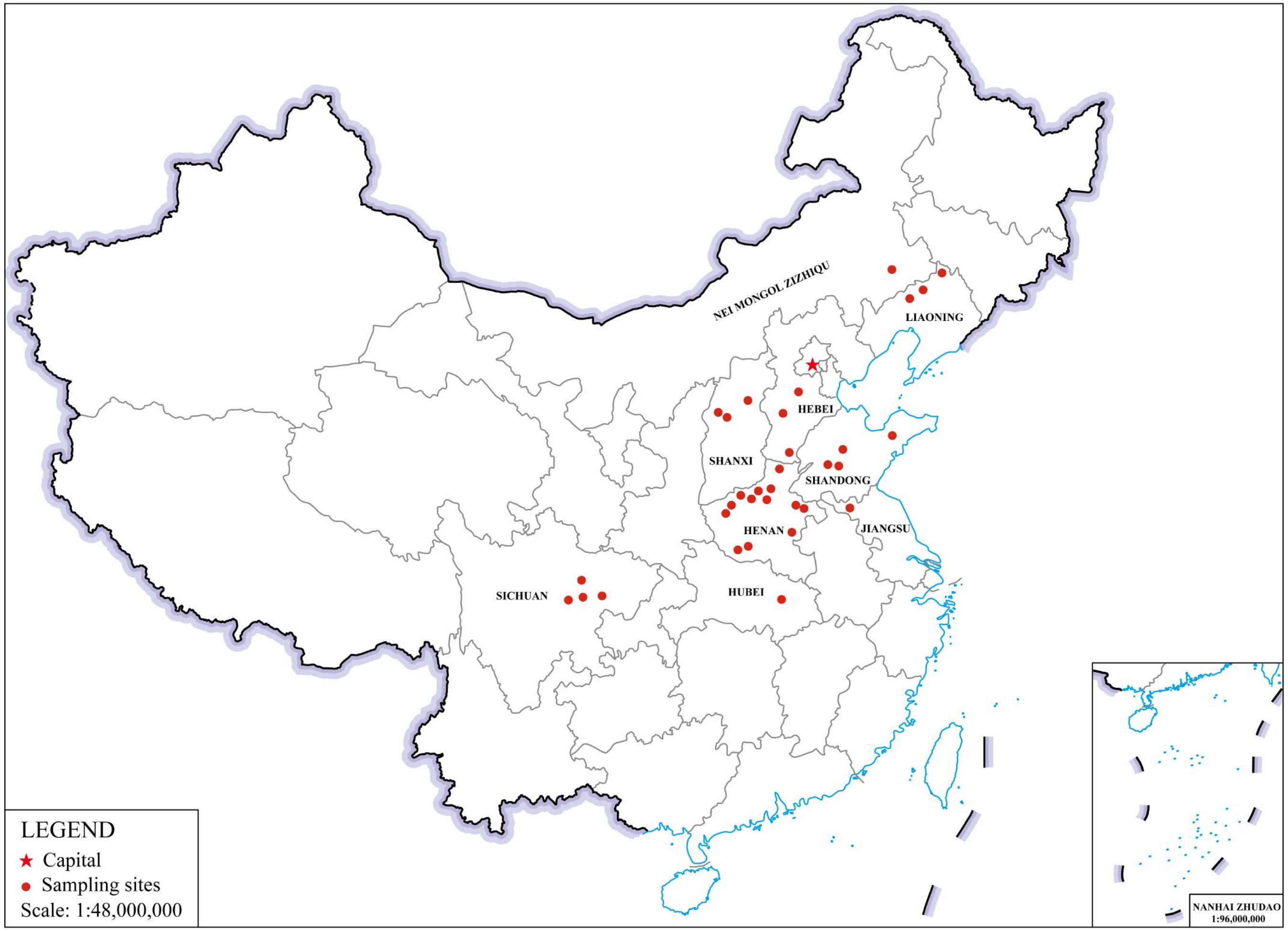

Supplementary Figure S1. Sampling sites of the 41 *D. arachidicola* isolates used in this study in China. The map was modified based on map GS(2019)1673.

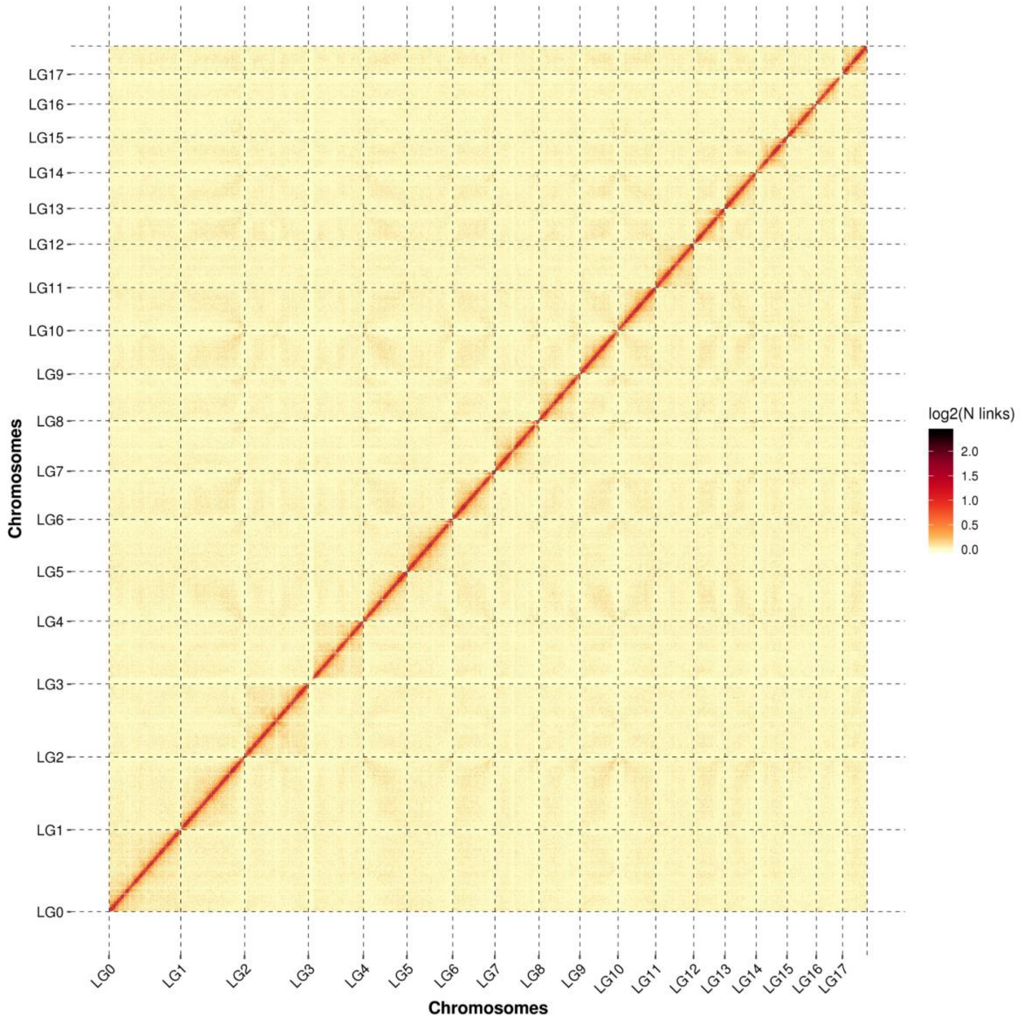

Supplementary Figure S2. Heat map of chromosomal interaction based on Hi-C assembly. “LG” means Lachesis group.

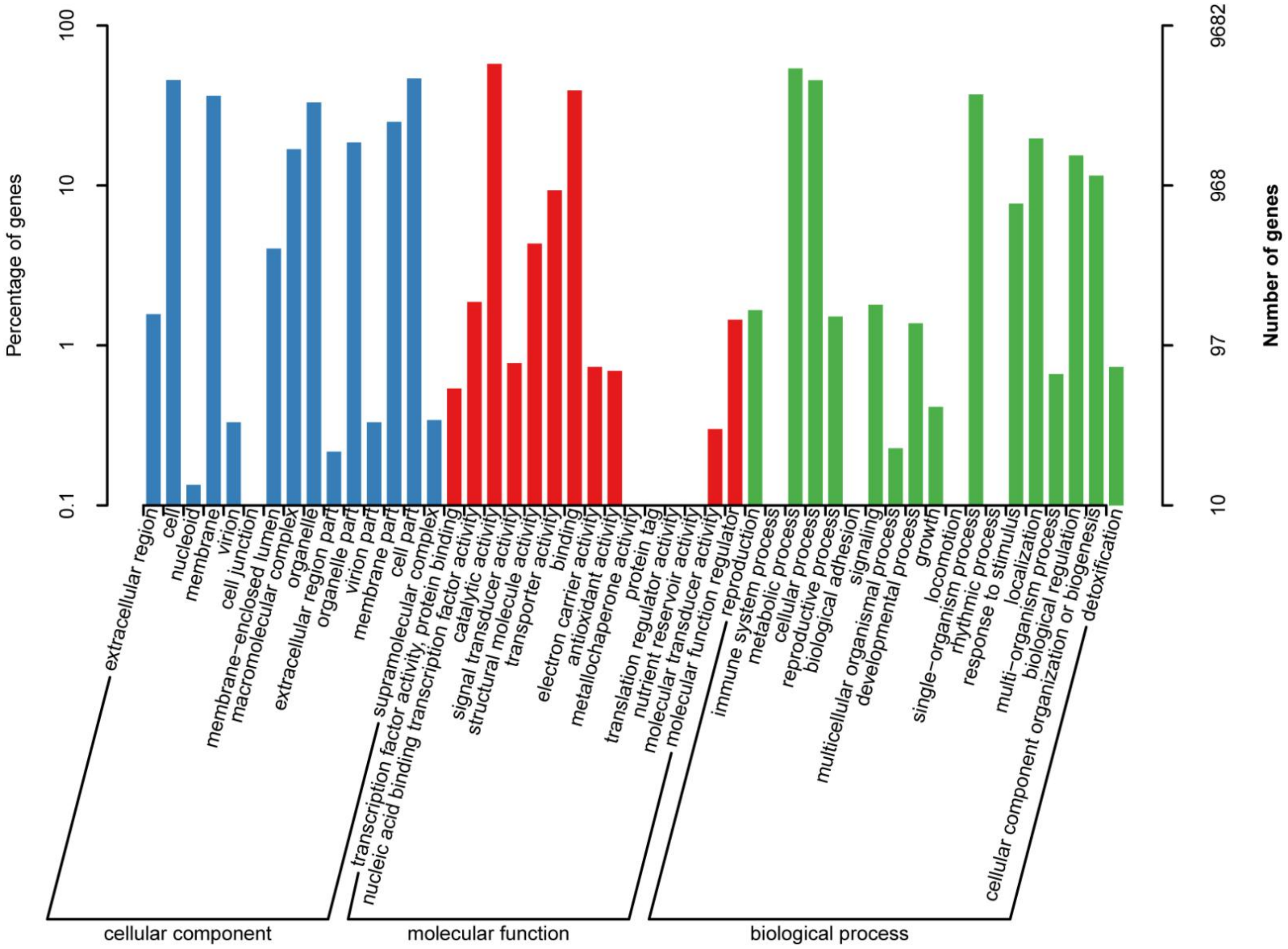

Supplementary Figure S3. Category statistics for GO annotation of *D. arachidicola* genes.

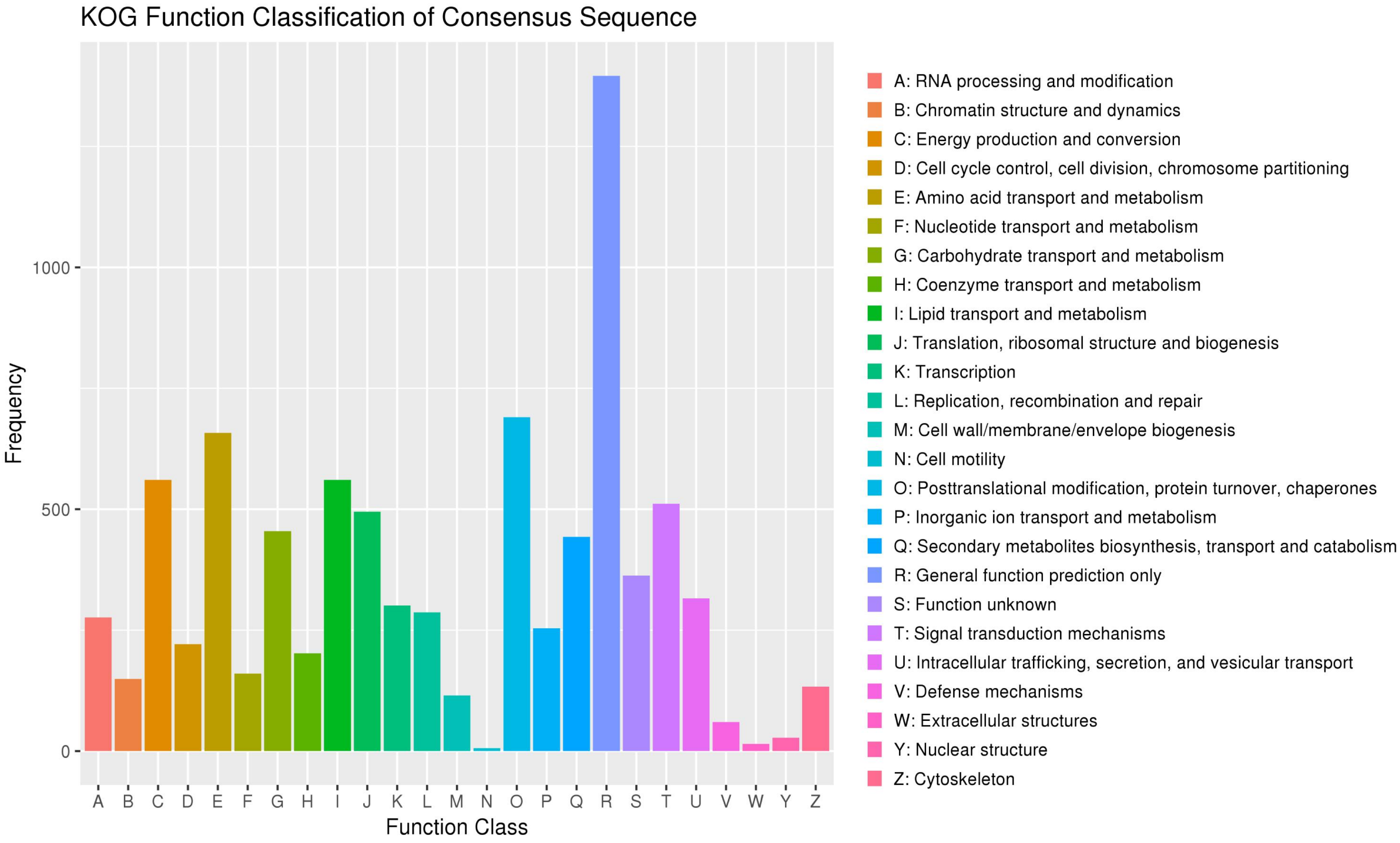

Supplementary Figure S4. Category statistics for KOG annotation of *D. arachidicola* genes.

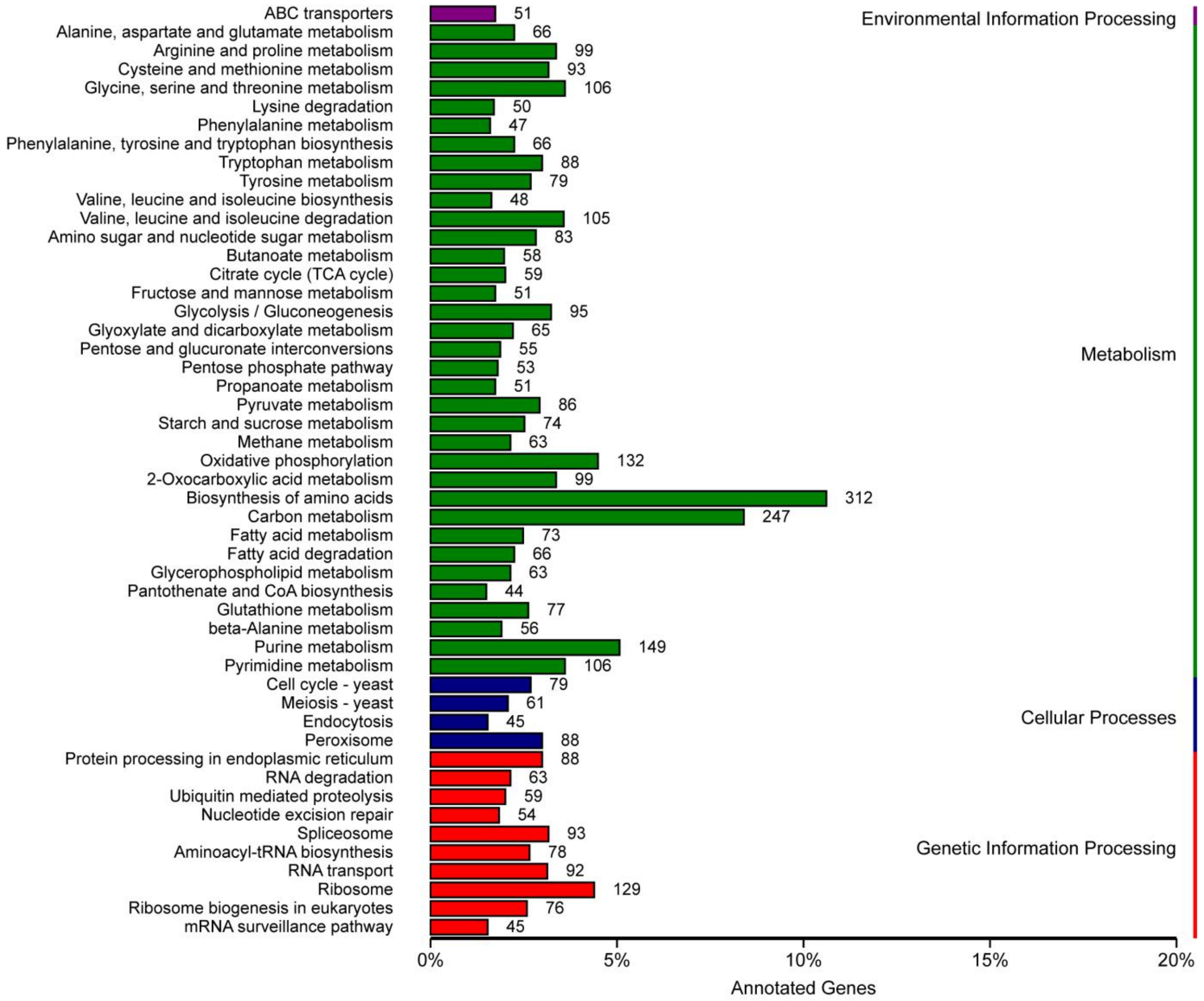

Supplementary Figure S5. Category statistics for KEGG annotation of *D. arachidicola* genes.

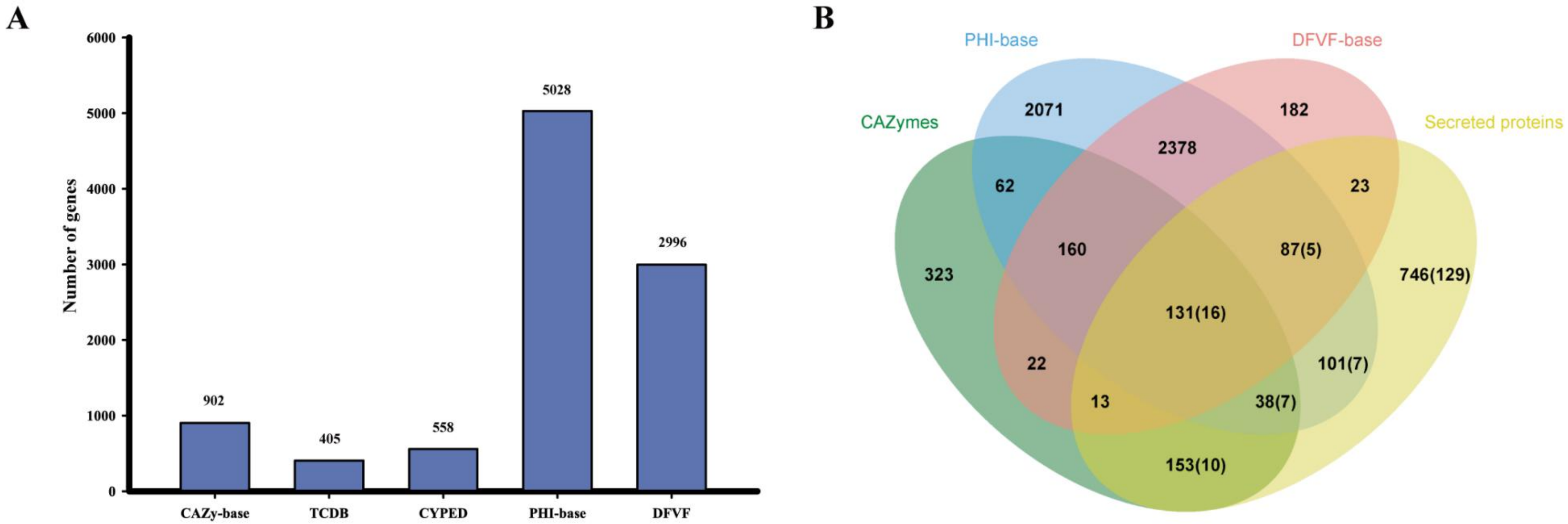

Supplementary Figure S6. The functional annotations of *D. arachidicola*. (A) The number of genes that have hits in different databases. (B) Venn diagram based on annotated genes in CAZy-base, PHI-base, DFVF, and genes of predicted secreted proteins; the numbers in parentheses represent the number of predicted effector proteins.

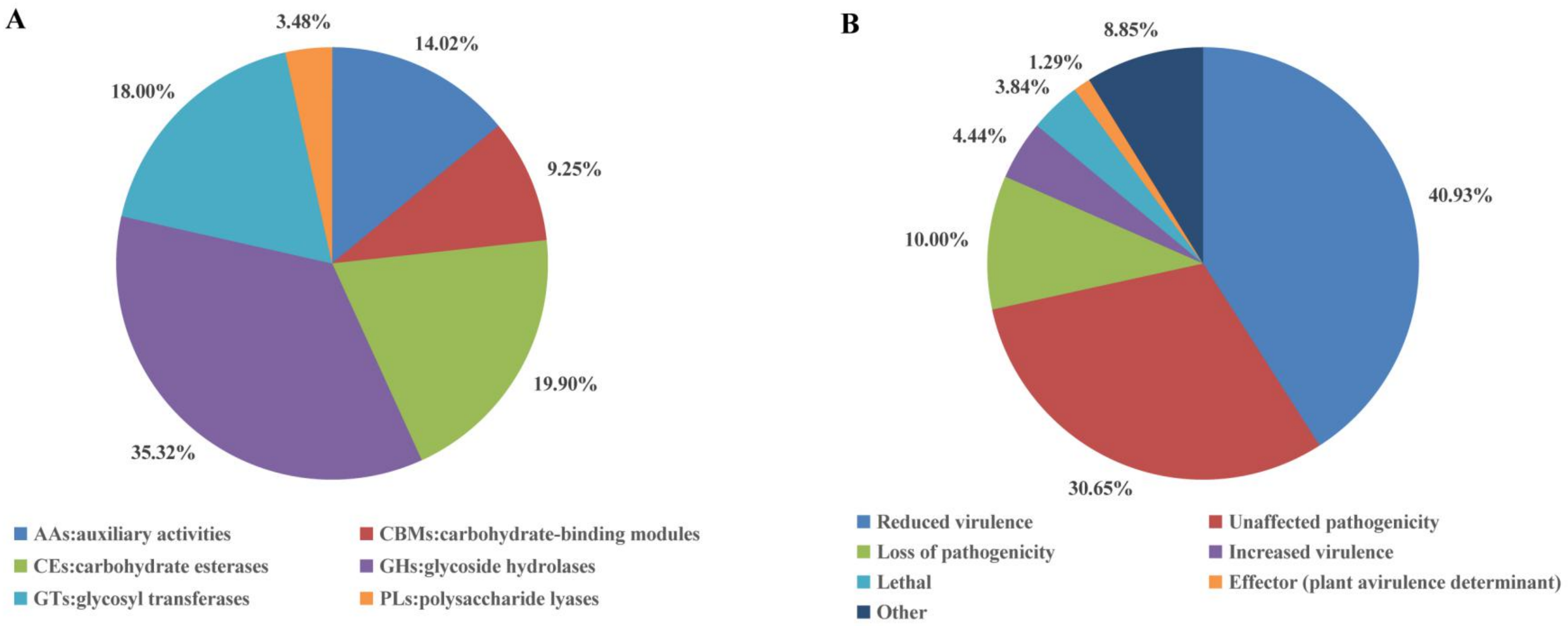

Supplementary Figure S7. Percentage of genes belonged to different categories. (A) Percentage of genes that have hits in different families of CAZyme. (B) Percentage of genes with different PHI phenotypes.

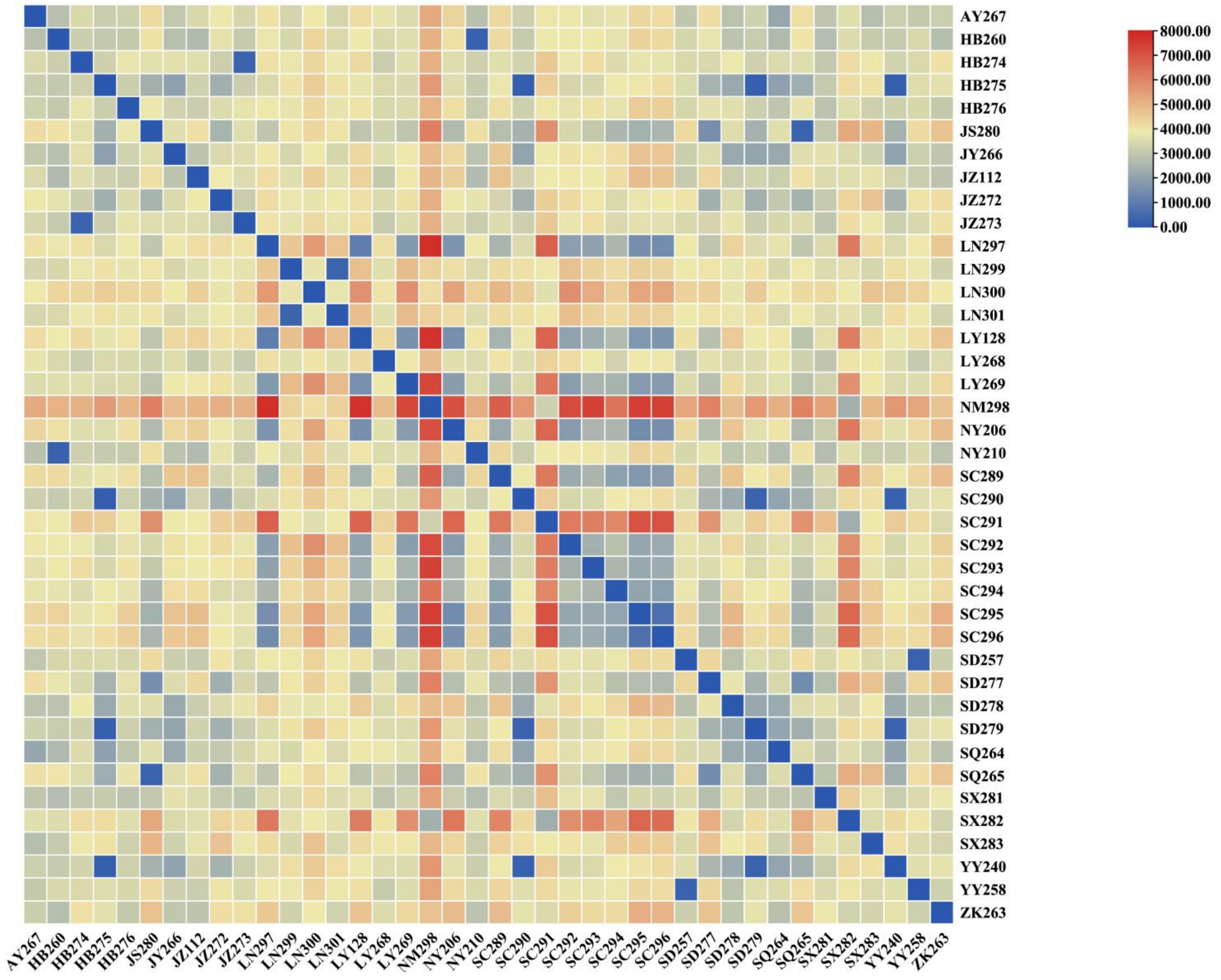

Supplementary Figure S8. Intraspecific comparison of variations of *D. arachidicola* isolates. Results visualized by TBtools v1.098769.
