# Supplementary material for: Intraspecific Comparative Analysis Reveals Genomic Variation of Didymella arachidicola and Pathogenicity Factors Potentially Related to Lesion Phenotype
Source: Biology (Basel). 2023 Mar 21;12(3):476. doi: 10.3390/biology12030476 (PMC10045276; doi:10.3390/biology12030476)
Supplement: Supplementary file 1 [file biology-12-00476-s001.zip › Supplementary Tables.pdf]

Supplementary Tables

Intraspecific Comparative Analysis Reveals Genomic Variation of *Didymella arachidicola* and Pathogenicity Factors Potentially Related to Lesion Phenotype

Shaojian Li *,†, Zhenyu Wang *,†, Meng Gao, Tong Li, Xiaowei Cui, Junhuai Zu, Suling Sang, Wanwan Fan and Haiyan Zhang

Institute of Plant Protection, Henan Key Laboratory of Crop Pest Control, International Joint Research Laboratory for Crop Protection of Henan, Key Laboratory of Integrated Pest Management on Crops in Southern Region of North China, Henan Academy of Agricultural Sciences, Zhengzhou 450000, China

* Correspondence: lishaojianli@126.com (S.L.); wangzy21@163.com (Z.W.)

Supplementary Table S1. The gene numbers predicted by different software

Strategy	Software	Homology species	Gene number
Ab initio prediction	Augustus		12,728
	Genscan		12,139
	GeneID		16,627
	GlimmerHMM		15,287
	SNAP		16,061
Homology based	GeMoMa	*Didymella exigua*	12,035
		Didymella heteroderae	10,499
		Didymella keratinophila	10,549
RNA-seq based	PASA		14,743
	TransDecoder		21,683
Integration	EVM		16,629

Supplementary Table S2. The statistics of gene annotation

Supplementary Table S4. All identified SNPs, InDels and filtered high-quality SNPs

Supplementary Table S6. The integrated gene set that shows the pathogenicity-related genes

Supplementary Table S2, S4, and S6 are listed as excel files due to their large size.

Supplementary Table S3. Summary of re-sequenced isolates, mapping and variants relative to the reference genome

Isolates	Origins	Clean reads	Q30 (%)	GC content (%)	Mapping rate (%)	SNPs	Ti/Tv	InDels -Insertion	InDels -Deletion
AY267	Anyang, Henan	5,227,713	95.21	52.68	98.28	3,417	3.13	141	150
HeB274	Baoding, Hebei	4,557,946	95.59	52.70	97.55	2,959	3.07	131	136
HeB275	Handan, Hebei	4,454,868	95.10	52.08	98.50	1,958	3.12	86	119
HeB276	Shijiazhuang, Hebei	4,447,879	95.31	52.81	99.02	3,251	3.09	163	158
HuB260	Wuhan, Hubei	3,010,100	93.92	52.58	99.41	3,214	3.07	140	148
JS280	Xuzhou, Jiangsu	4,530,529	95.36	52.23	98.67	2,679	3.47	129	139
JY266	Jiyuan, Henan	4,789,405	95.19	52.54	98.15	2,358	3.27	95	109
JZ112	Jiaozuo, Henan	4,319,063	94.58	52.28	97.01	3,293	3.23	147	186
JZ272	Jiaozuo, Henan	4,706,199	94.70	52.68	93.78	2,472	3.09	120	139
JZ273	Jiaozuo, Henan	4,524,079	94.21	52.72	94.46	2,921	3.13	138	160
LN297	Fuxin, Liaoning	3,936,144	94.17	51.44	97.73	3,554	3.27	177	172
LN299	Shenyang, Liaoning	3,190,707	94.54	51.05	98.85	3,461	3.14	163	174
LN300	Tieling, Liaoning	3,293,057	94.76	51.21	97.22	3,906	3.28	188	173
LN301	Tieling, Liaoning	3,269,704	94.72	51.47	98.49	3,493	3.21	169	173
LY128	Luoyang, Henan	2,767,415	93.65	52.70	99.66	3,993	3.40	217	190
LY268	Luoyang, Henan	4,764,516	94.50	52.13	92.46	2,901	3.13	153	149
LY269	Luoyang, Henan	4,583,975	94.31	52.81	95.17	3,565	3.39	163	179
NM298	Tongliao, Neimeng	4,043,814	94.36	51.57	93.69	5,374	3.25	267	229
NY206	Nanyang, Henan	2,820,889	94.11	52.74	99.30	3,157	3.30	177	168

NY210	Nanyang, Henan	4,512,676	94.37	52.24	97.41	3,210	2.99	130	153
SC289	Chengdu, Sichuan	3,552,798	94.02	51.86	98.36	3,580	3.33	187	174
SC290	Chengdu, Sichuan	3,373,635	93.65	51.31	98.69	1,986	2.97	89	101
SC291	Deyang, Sichuan	3,510,557	96.08	51.20	98.70	4,854	3.17	202	218
SC292	Deyang, Sichuan	3,368,239	95.29	51.41	98.56	3,700	3.28	190	163
SC293	Chengdu, Sichuan	3,415,390	95.24	50.95	97.99	3,614	3.20	178	185
SC294	Chengdu, Sichuan	3,333,554	93.83	51.32	98.29	3,140	3.20	177	153
SC295	Nanchong, Sichuan	3,572,399	93.57	51.58	98.38	3,541	3.28	181	165
SC296	Nanchong, Sichuan	3,909,250	94.10	51.59	98.58	3,465	3.30	166	172
SD257	Laixi, Shandong	2,339,160	91.78	53.03	99.16	3,707	2.91	207	173
SD277	Taian, Shandong	3,531,785	94.13	51.88	95.72	2,301	3.17	113	190
SD278	Taian, Shandong	4,227,415	94.58	51.96	90.13	3,103	3.37	146	170
SD279	Jinan, Shandong	4,037,187	94.35	52.13	93.75	2,045	3.17	100	131
SQ264	Shangqiu, Henan	4,440,903	94.42	52.24	98.15	2,457	3.23	97	161
SQ265	Shangqiu, Henan	4,435,473	95.23	52.08	98.87	2,597	3.23	122	142
SX281	Xinzhou, Shanxi	4,546,091	95.18	52.68	94.94	2,442	3.14	138	130
SX282	Lvliang, Shanxi	4,344,845	95.38	52.26	98.71	4,554	3.26	198	177
SX283	Lvliang, Shanxi	4,268,001	94.22	51.88	97.54	4,059	3.41	181	211
YY240	Xinxiang, Henan	5,359,735	95.66	52.48	98.50	2,024	3.05	99	115
YY258	Xinxiang, Henan	4,586,621	95.17	52.06	99.12	3,455	3.05	158	128
ZK263	Zhoukou, Henan	4,976,040	95.44	52.65	98.26	3,907	3.25	164	147

Supplementary Table S5. The statistics of differential genes

Isolates	Non-synonymous SNPs in exons	Genes with non-synonymous SNP [1]	InDels in exons	Genes with CDS-InDel	Genes in differential gene set
AY267	381	314	42	30	254
HeB274	325	286	27	27	227
HeB275	224	167	27	22	137
HeB276	367	292	49	38	247
HuB260	366	305	32	29	244
JS280	298	241	43	37	204
JY266	228	185	22	19	157
JZ112	347	295	52	43	248
JZ272	290	229	41	30	196
JZ273	320	282	29	29	223
LN297	380	300	53	33	251
LN299	367	311	42	32	256
LN300	429	340	59	49	291
LN301	368	311	47	37	256
LY128	397	319	46	33	255
LY268	308	270	34	29	216
LY269	381	329	40	32	264
NM298	497	385	56	47	315

NY206	334	283	48	33	231
NY210	356	309	35	32	249
SC289	394	315	56	43	266
SC290	239	178	30	23	146
SC291	507	420	51	41	331
SC292	381	291	49	33	238
SC293	393	300	56	37	256
SC294	360	311	37	33	258
SC295	390	327	44	34	265
SC296	371	315	48	33	257
SD257	351	292	59	39	244
SD277	255	215	55	51	199
SD278	326	262	42	33	222
SD279	242	176	33	28	148
SQ264	258	202	48	36	174
SQ265	292	234	43	34	200
SX281	297	256	62	50	227
SX282	474	402	54	40	326
SX283	368	294	53	38	243
YY240	239	181	31	25	151
YY258	350	289	40	27	227
ZK263	389	320	40	30	254

[1] Repeat statistics were not performed for multiple non-synonymous SNPs in the same gene

Supplementary Table S7. The repository and accession numbers of the datasets presented in this study

Isolates	Organism	Repository	Accession Numbers	
			Bioproject	Assembly / SRA
YY187	*Didymella arachidicola*	NCBI	PRJNA562378	GCA_016630955.2
AY267	*Didymella arachidicola*	NCBI	PRJNA562378	SRR22426688
HeB274	*Didymella arachidicola*	NCBI	PRJNA562378	SRR22426687
HeB275	*Didymella arachidicola*	NCBI	PRJNA562378	SRR22426676
HeB276	*Didymella arachidicola*	NCBI	PRJNA562378	SRR22426665
HuB260	*Didymella arachidicola*	NCBI	PRJNA562378	SRR22426654
JS280	*Didymella arachidicola*	NCBI	PRJNA562378	SRR22426653
JY266	*Didymella arachidicola*	NCBI	PRJNA562378	SRR22426652
JZ112	*Didymella arachidicola*	NCBI	PRJNA562378	SRR22426651
JZ272	*Didymella arachidicola*	NCBI	PRJNA562378	SRR22426650
JZ273	*Didymella arachidicola*	NCBI	PRJNA562378	SRR22426649
LN297	*Didymella arachidicola*	NCBI	PRJNA562378	SRR22426686
LN299	*Didymella arachidicola*	NCBI	PRJNA562378	SRR22426685
LN300	*Didymella arachidicola*	NCBI	PRJNA562378	SRR22426684
LN301	*Didymella arachidicola*	NCBI	PRJNA562378	SRR22426683
LY128	*Didymella arachidicola*	NCBI	PRJNA562378	SRR22426682
LY268	*Didymella arachidicola*	NCBI	PRJNA562378	SRR22426681
LY269	*Didymella arachidicola*	NCBI	PRJNA562378	SRR22426680
NM298	*Didymella arachidicola*	NCBI	PRJNA562378	SRR22426679

NY206	*Didymella arachidicola*	NCBI	PRJNA562378	SRR22426678
NY210	*Didymella arachidicola*	NCBI	PRJNA562378	SRR22426677
SC289	*Didymella arachidicola*	NCBI	PRJNA562378	SRR22426675
SC290	*Didymella arachidicola*	NCBI	PRJNA562378	SRR22426674
SC291	*Didymella arachidicola*	NCBI	PRJNA562378	SRR22426673
SC292	*Didymella arachidicola*	NCBI	PRJNA562378	SRR22426672
SC293	*Didymella arachidicola*	NCBI	PRJNA562378	SRR22426671
SC294	*Didymella arachidicola*	NCBI	PRJNA562378	SRR22426670
SC295	*Didymella arachidicola*	NCBI	PRJNA562378	SRR22426669
SC296	*Didymella arachidicola*	NCBI	PRJNA562378	SRR22426668
SD257	*Didymella arachidicola*	NCBI	PRJNA562378	SRR22426667
SD277	*Didymella arachidicola*	NCBI	PRJNA562378	SRR22426666
SD278	*Didymella arachidicola*	NCBI	PRJNA562378	SRR22426664
SD279	*Didymella arachidicola*	NCBI	PRJNA562378	SRR22426663
SQ264	*Didymella arachidicola*	NCBI	PRJNA562378	SRR22426662
SQ265	*Didymella arachidicola*	NCBI	PRJNA562378	SRR22426661
SX281	*Didymella arachidicola*	NCBI	PRJNA562378	SRR22426660
SX282	*Didymella arachidicola*	NCBI	PRJNA562378	SRR22426659
SX283	*Didymella arachidicola*	NCBI	PRJNA562378	SRR22426658
YY240	*Didymella arachidicola*	NCBI	PRJNA562378	SRR22426657
YY258	*Didymella arachidicola*	NCBI	PRJNA562378	SRR22426656
ZK263	*Didymella arachidicola*	NCBI	PRJNA562378	SRR22426655
